# Lung Macrophages: Multifunctional Regulator Cells for Metastatic Cells

**DOI:** 10.3390/ijms20010116

**Published:** 2018-12-29

**Authors:** Naofumi Mukaida, Takuto Nosaka, Yasunari Nakamoto, Tomohisa Baba

**Affiliations:** 1 Division of Molecular Bioregulation, Cancer Research Institute, Kanazawa University Kakuma-machi, Kanazawa, Ishikawa 920-1192, Japan; nosat@u-fukui.ac.jp (T.N.), sergenti@staff.kanazawa-u.ac.jp (T.B.); 2 Second Department of Internal Medicine, Faculty of Medical Sciences, University of Fukui, Yoshida-gun, Fukui 910-1193, Japan; nakamoto-med2@med.u-fukui.ac.jp

**Keywords:** alveolar macrophage, classical monocyte, interstitial macrophage, metastasis-associated macrophage, patrolling monocyte, perivascular macrophage

## Abstract

Metastasis is responsible for most of the cancer-associated deaths and proceeds through multiple steps. Several lines of evidence have established an indispensable involvement of macrophages present at the primary tumor sites in various steps of metastasis, from primary tumor growth to its intravasation into circulation. The lungs encompass a large, dense vascular area and, therefore, are vulnerable to metastasis, particularly, hematogenous ones arising from various types of neoplasms. Lung tissues constitutively contain several types of tissue-resident macrophages and circulating monocytes to counteract potentially harmful exogenous materials, which directly reach through the airway. Recent advances have provided an insight into the ontogenetic, phenotypic, and functional heterogeneity of these lung macrophage and monocyte populations, under resting and inflammatory conditions. In this review, we discuss the ontogeny, trafficking dynamics, and functions of these pulmonary macrophages and monocytes and their potential roles in lung metastasis and measures to combat lung metastasis by targeting these populations.

## 1. Introduction

Metastatic lesions are presumed to be responsible for nearly 90% of cancer-associated deaths [1]. Metastasis is defined as the dissemination of cancer cells from the primary tumor site to distant organs, resulting in the formation of secondary tumor tissues [1,2]. Two models, namely linear progression and parallel progression models, have been proposed to explain the molecular mechanisms of metastasis [3]. In the linear progression model, metastasis is a late event arising from the accumulation of genetic mutations in cancer cells, during the course of tumor progression and consequently cancer cells at metastatic sites have more mutated neoantigens than those at primary sites [2]. Thus, cancer cells at metastasis sites might more efficiently induce innate and acquired immune response, but this response might be counteracted by tumor-associated macrophages (TAMs), macrophages present at tumor sites, with a potential capacity to exert immune suppressive activities in most cases [4]. According to the parallel progression model, cancer cells can undergo genetic evolution independently at primary and metastatic sites and the numbers of neoantigens may not be different between cancer cells at primary and metastatic sites. Parallel progression model is supported by observations in mouse models, where cancer cells disseminated at an early time point without appreciable primary tumor formation [5,6,7]. However, comparative genomic studies of human primary tumors and metastases indicates the coexistence of elements of both linear and parallel metastatic progression models even within same patients [3]. Thus, it is an open question regarding the vulnerability of metastatic cancer cells to innate immune memory.

**Figure 1:**
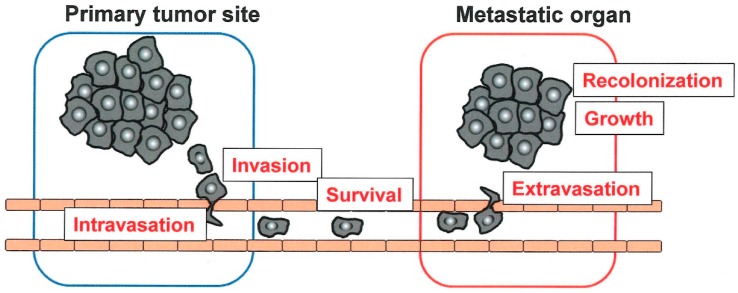
Multiple steps of metastasis. Tumor grows at primary site and invades the surrounding tissues to intravasate into circulation. Tumor cells, which survive in circulation, extravasate and recolonize a target organ to grow there.

Metastasis proceeds through multiple steps. Cancer cells at the primary site locally invade the surrounding tissues, intravasate into systemic circulation including blood and lymphatics, migrate through circulation, and extravasate through vascular walls into the parenchyma of distant organs to seed. The seeding tumor cells form micrometastatic colonies and subsequently proliferate into overt and clinically detectable metastatic lesions (Figure 1) [1,2]. During metastasis, specific phenotypic changes in tumor cells such as epithelial-mesenchymal transition (EMT), play an important role. This process is also profoundly affected by the interaction with the host microenvironment, particularly when tumor cells reach distant organs to recolonize [8].

A characteristic feature of metastasis is organ tropism, a predilection of metastasis to specific organs. The lung is the second most common site for the occurrence of metastasis [8]. A predilection of metastasis to the lungs has been noted in several types of cancers including breast, kidney, melanoma, and sarcoma [9]. Other types of tumors such as colon, bladder, head-and-neck, and pancreatic cancers, often, but not preferentially, metastasize to the lungs. More than 100 years ago, Paget reported that breast cancer metastasized frequently to specific organs such as lungs and bone and proposed the “seed and soil” hypothesis [10]. Paget claimed that metastasis was not due to a chance event, but rather that some tumor cells (the seed) could grow preferentially in select organs (the soil), which could provide the seed with optimal microenvironment for its growth [10]. Organ tropisms, however, can also be explained by the anatomical/mechanical hypothesis, which underscores the anatomical roles of the vascular and lymphatic drainage from the primary tumor site. This is exemplified by tumors arising in the gastrointestinal tract with a propensity to metastasize through a hematogenous route to the liver, which has a unique venous drainage, taking place through the portal venous system [8]. The high frequency of lung metastasis can be partly ascribed to a large, dense vascular area in the lung, which is estimated to be as much as 100 m^2^ and is significantly higher than that of any other organs [9]. Moreover, cancer cells are predisposed to be arrested in capillary beds in the lungs, because the diameter of cancer cells (20 μm) is five times larger than that of lung capillaries (4 μm) [8]. Thus, under clinical situations “seed and soil” and anatomical/mechanical hypotheses are not mutually exclusive and either or both can be operational during metastasis, depending on the types of tumors and clinical scenarios.

In the following sections, we will discuss the ontogeny and functions of lung monocytes and macrophages, and their roles in the process of lung metastasis. Most of the studies discussed were conducted on mouse metastatic models, unless indicated otherwise.

## 2. Monocytes and Tissue-Resident Macrophages in the Normal Lungs; Ontogeny and Functions

Normal lung tissues constitutively contain abundant blood monocytes and tissue-resident macrophages. Monocytes can be classified into two subsets, classical (inflammatory) and patrolling monocytes, and lung tissue-resident macrophages consist of two distinct populations, alveolar macrophages (AMs) and interstitial macrophages (IMs). These cell populations can be discriminated based on the expression patterns of cell surface markers (Table 1) [11,12].

Classical monocytes traffic into inflamed tissues in response to locally-produced CCL2 and secrete cytokines, chemokines, and nitric oxide (NO) [13]. These cells sometimes polarize into so-called tip-dendritic cells (DCs) with a capacity to present antigen and express tumor necrosis factor (TNF)-α and NO synthase [14]. Moreover, these classical monocytes move to perivascular areas of tumor tissues to become so-called metastasis-associated macrophages (MAMs) with characteristic IM phenotypes [15,16], as discussed in the following sections. Patrolling monocytes are generated depending on the orphan nuclear factor, Nr4a1 [17], and these cells continuously inspect the vascular system to remove dead and stressed endothelium in collaboration with neutrophils [18]. Moreover, immunosuppressive activity is assigned to patrolling monocytes [19].

The presence of another distinct type of monocytes has been reported in the lungs [20]. These are Ceacam1^+^Msr1^+^Ly6C^−^F4/80^−^Mac1^+^monocytes with a segregated nucleus and exhibit granulocytic characteristics. Moreover, they require CCAAT/enhancer binding protein β (C/EBPβ) for their development from their Ly6C^−^FcεRI^+^ granulocyte/macrophage progenitors and are critical for lung fibrosis. However, at present, the role of these atypical monocytes under tumor-bearing conditions including lung metastatic process, remains elusive.

Macrophages are phagocytes present in all tissues and are polarized into two phenotypically and functionally distinct states, M1 and M2, under the influence of surrounding microenvironment [21]. M1 macrophages are polarized by Th1 cytokines including interferon (IFN)-γ and tumor necrosis factor (TNF)-α and produce pro-inflammatory cytokines such as TNF-α, interleukin (IL)-1α, IL-1β, IL-6, and cyclooxygenase (COX)-2. Additionally, M1 macrophages abundantly express inducible NO synthetase to generate reactive oxygen species (ROS). As a result, M1 macrophages exhibit pro-inflammatory, anti-microbial, and anti-tumoral activity. In contrast, M2 macrophages are induced by IL-10 and IL-33 as well as Th2 cytokines such as IL-4 and IL-13. M2 macrophages express arginase-1 and produce anti-inflammatory cytokines including IL-10 and transforming growth factor (TGF)-β. However, these phenotypes are plastic and reversible. Exposure of M2 macrophages to M1 signals, or vice versa, can induce re-polarization or reprogramming of polarized macrophages [21]. Moreover, tissue-resident macrophages can exhibit either phenotype, depending on the signals provided by their surrounding microenvironment.

Two distinct types of tissue-resident macrophages are localized differentially in lung tissues. AMs are located on the luminal surface but are closely adherent to the alveolar epithelium and are directly exposed to air and the environment [22]. Consequently, AMs are the first to encounter incoming pollutants and pathogens and help organize the initiation and resolution of the inflammatory and immune responses in the lungs. AMs also have an indispensable role in the maintenance of airspace by clearing the surfactant that lines the surface of the alveoli [22]. IMs comprise 30 to 40% of lung macrophages and are located in the lung tissue interstitium, particularly in the vicinity of bronchi [23]. IMs are presumed to remodel tissue and to influence DC functions to prevent airway allergy [24]. Differences in the localization cannot always distinguish between AMs and IMs, since specific immune stimuli induced the infiltration of cells with an IM-like phenotype into the airway lumen [25]. Thus, AMs and IMs are discriminated based on the distinct patterns of several surface markers and these patterns differ between mouse and human (Table 1) [23].

**Figure 2:**
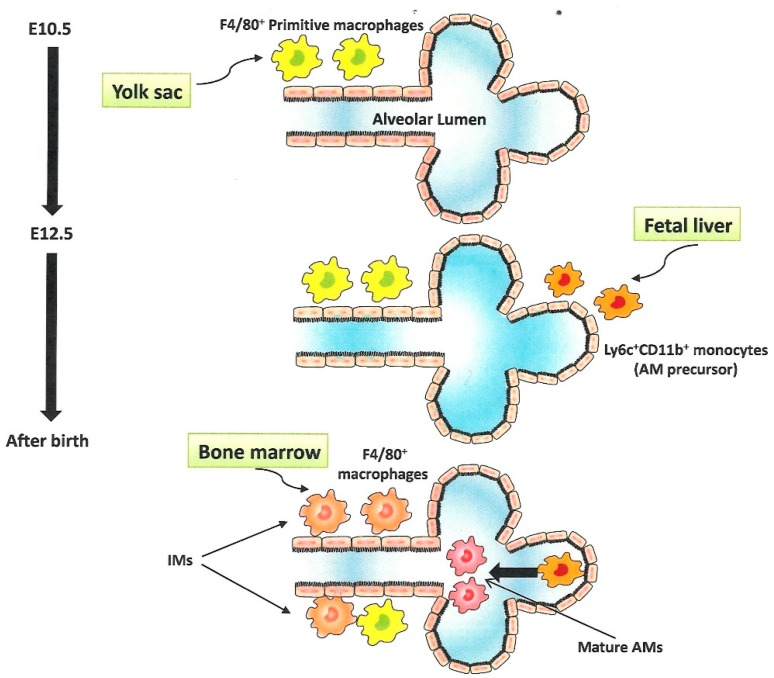
Ontogeny of alveolar macrophages (AMs) and interstitial macrophages (IMs). Beginning at embryonic day (E) 10.5, yolk sac-derived primitive macrophages migrate into lung parenchyma. Beginning at E 12.5, fetal liver-derived monocytes migrate into lung parenchyma and persist as AM precursors. After birth, AM precursors migrate into alveolar lumen and differentiate into mature AMs under the guidance of GM-CSF. Concurrently, bone marrow-derived macrophages migrate into parenchyma to replace some of the yolk sac-derived primitive macrophages and differentiate into IMs. Persistent yolk sac-derived macrophages also differentiate into IMs.

Historically, all tissue-resident macrophages were presumed to arise from circulating bone marrow-derived monocytes [26]. However, this hypothesis was challenged by a seminal study which identified yolk sac as another source of macrophages which is active before definitive hematopoiesis begins in the fetal liver [27]. This assertion was further strengthened by a subsequent detailed fate mapping study [28]. Moreover, Guilliams et al. demonstrated that yolk sac-derived F4/80^high^CD11b^l^°^w^ primitive macrophages and fetal liver-derived Ly6C^high^CD11b^high^ monocytes sequentially colonized in the developing lungs beginning at embryonic day (E) 10.5 and 12.5, respectively [29]. However, only fetal liver-derived monocytes enter the alveolar lumen and differentiate into mature AMs after birth under the guidance of the alveolar niche. These observations were further corroborated by a study using genetic lineage tracing and parabiosis [30]. Yolk sac-derived primitive macrophages localize to the peripheral and perivascular regions and persist as primitive IMs even at birth. After birth, bone marrow-derived F4/80^+^ macrophages enter the lung parenchyma to replace a substantial portion of yolk sac-derived primitive macrophages in the central areas. Subsequently, both residual primitive IMs in peripheral regions and bone marrow-derived F4/80^+^ macrophages differentiate into IMs [30] (Figure 2). 

The terminal differentiation of AMs however, requires the alveolar niche which appears only after birth [22]. The most notable niche is type II alveolar epithelial cells, which produce granulocyte macrophage (GM)-colony stimulating factor (GM-CSF)/CSF2. Consistently, *GM-CSF/CSF2* deficiency leads to alveolar proteinosis with enhanced susceptibility to respiratory infection. These defects are associated with impairment of terminal differentiation of AMs [31] and reduced expression of the transcription factor PU.1 [32]. Selective GM-CSF/CSF2 expression in lungs induced the terminal differentiation of AMs in a PU.1-dependent manner and rescued the pathological changes observed in *GM-CSF/CSF2*-deficient mice [32]. In addition to type II alveolar epithelial cells, basophils present near the alveoli can be a source of GM-CSF/CSF2 [33].

A recent study has revealed an indispensable role of von Hippel-Lindau protein (VHL), a negative regulator of hypoxia-inducible factors (HIFs), in the terminal differentiation of AMs [34]. Self-renewal capacity was severely impaired in AMs that were selectively depleted of *Vhl* gene. Moreover, *Vhl*-deficient AMs failed to reverse pulmonary alveolar proteinosis when transplanted into *GM-CSF/CSF2 receptor (R)*-deficient mice [34]. Thus, intact oxygen-sensing capacity is required for the terminal differentiation of AMs in addition to GM-CSF/CSF2-mediated signals. AMs also require another transcription factor, peroxisome proliferator-activated receptor (PPAR)-γ for the maintenance of surfactant homeostasis [35].

Although AMs can maintain their population through in situ self-renewal in steady states, they can be replenished from circulating monocytes under stressed conditions such as irradiation and infection [29]. During lung fibrosis circulating monocytes infiltrate into lungs and persist for a long time with similar phenotypes as observed on AMs [36]. Likewise, infection with murid herpes virus caused the replacement of resident AMs by blood-derived classical Ly6C^high^ monocytes, which differentiated into AMs with a capacity to block the ability of DCs to trigger allergic response by Th2 cells [37]. AM numbers in mixed bone marrow chimeric mice were decreased markedly after an intranasal instillation of clodronate liposome (CLL) but recovered fully around one month after the treatment, at which point nearly half of AMs were replaced with donor-derived cells [38]. Moreover, no substantial differences were observed at either the transcriptional or functional levels between recipient- and donor-derived AMs in these bone marrow chimeric mice [38]. These observations would indicate that bone marrow contains AM precursors, which can migrate through circulating blood into lung to differentiate into mature AMs. Likewise, we observed in a mouse lung metastasis model that blood-derived AM precursors can infiltrate into lungs to differentiate into AMs [39], as discussed in the following section. However, it remains elusive on the precise phenotypes of AM precursors in bone marrow and circulating blood.

## 3. Monocytes and Macrophages in the Lung with Metastatic Tumor Cells

Patrolling monocytes have recently emerged as players in lung metastasis [40]. These monocytes interacted with metastasizing tumor cells, removed tumor materials from the lung vascular system and promoted natural killer (NK) cell recruitment and activation. *Nr4a1* gene deletion leads to a selective deficiency of patrolling monocytes in mice [40]. Due to the capacity of patrolling monocytes to counteract, *Nr4a1*-deficient mice devoid of patrolling monocytes were prone to develop metastasis in several models [40]. However, additional studies are required to clarify the precise roles of patrolling monocytes in lung metastasis.

**Figure 3:**
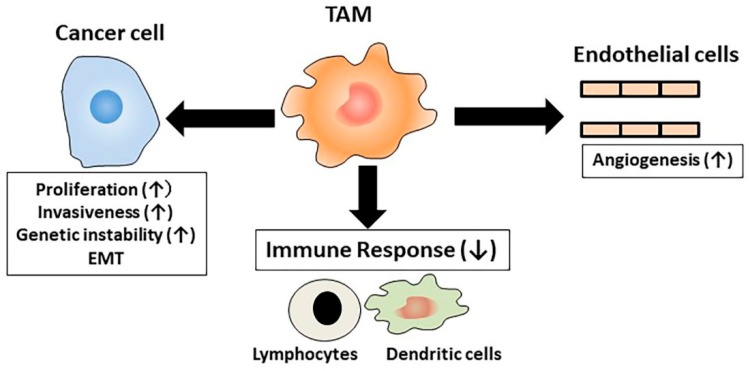
Effects of tumor-associated macrophage (TAM) on tumorigenesis. TAMs affect cancer cells by enhancing proliferation and invasiveness and inducing genetic instability and epithelial-mesenchymal transition (EMT). TAMs enhance angiogenesis and suppress tumor immunity.

Currently, classical monocytes are presumed to be a major source of TAMs, which are present in tumor tissues [41], although they can originate from monocytic myeloid-derived suppressor cells or tissue-resident macrophages. Macrophages can generally exert anti-tumor cytotoxicity in vitro and therefore, TAMs were originally presumed to be cytotoxic against tumor cells [4]. However, subsequent studies demonstrated that TAMs in most cancer tissues lack cytotoxic activities but exhibit M2-like phenotypes [21]. They further produce epidermal growth factor and vascular endothelial growth factor (VEGF), and inhibit immune responses [42]. As a consequence, TAMs can greatly impact almost all aspects of tumor progression at the primary site, including induction of angiogenesis, EMT, invasion, and proliferation, as well as providing a niche for cancer stem cells [41] (Figure 3). TAMs with a capacity to increase tumor extravasation and their seeding, are designated as MAMs [16]. MAMs expressed a marker of IM, CD11b and could be classified as IMs. However, in most studies on lung metastasis, TAMs in lung metastasis sites were identified solely based on the expression of F4/80 in mouse and that of CD64 in human and therefore, cannot be classified into IMs or AMs. Thus, we used TAMs when we discussed the paper which lacked the information on the detailed surface markers of macrophages.

TAMs exhibit a characteristic transcriptional program governed by a transcription factor, Ets2 [43]. Moreover, depletion of *Ets2* in TAMs decreased the frequency and size of lung metastases in three different mouse models using breast cancer cells. Furthermore, Ets2-expressing TAMs, displayed a gene signature consisting of 133 genes in human breast cancer expression data which retrospectively predicted the overall survival of breast cancer patients in two independent data sets [43]. TAMs expressed another transcription factor, E2f3, which is downstream of GM-CSF/CSF2. E2f3 regulated cytoskeleton rearrangements, cell migration and adhesion with few effects on cell proliferation [44]. Selective depletion of *E2f3* in TAMs but not in tumor cells, suppressed lung metastasis caused by murine breast cancer cells, without effects on the growth of primary tumors. These observations suggest the contribution of this transcription factor in lung metastasis.

Lung macrophages might sustain their M2-like phenotype due to the function of a G-protein-coupled receptor, Gpr132, which can detect lactate present abundantly in the acidic tumor microenvironment [45]. Gpr132 expression positively correlated with M2 macrophages, metastasis, and poor prognosis in patients with breast cancer. Moreover, Gpr132 deletion reduced M2 macrophages in lungs and impaired breast cancer metastasis [45].

Lung macrophages as well as tumor cells release a proteinase, cathepsin B, which can promote the proliferation of breast cancer colonies in the lung [46]. Another proteinase, matrix metalloproteinase (MMP)-9, was expressed by TAMs at the primary site of mouse breast cancer and contributed to subsequent lung metastasis [47]. Likewise, macrophages were recruited into lungs and expressed MMP-9 abundantly in response to a locally-produced macrophage-tropic chemokine CCL3, thereby promoting lung metastasis arising from an intravenous injection of murine renal adenocarcinoma cells [48]. MMP-9 expression in pre-metastatic lung endothelial cells and macrophages was found to be indispensable for lung metastasis in mouse models using either melanoma or lung carcinoma cells [49]. Moreover, primary tumor cells specifically induced MMP-9 expression in lung macrophages and endothelial cells by the action of VEGF receptor-1 (VEGF-R1). Blocking this receptor reduced MMP-9 expression and eventually lung metastasis, further indicating a crucial role of lung macrophages in lung metastasis [49].

Several pathways can be used for monocyte and/or macrophage recruitment into metastatic lungs. By acting on endothelin-1 (ET-1) receptor, tumor-derived ET-1 induced the migration of both tumor cells and macrophages into lungs and also induced the expression of pro-inflammatory cytokines including IL-6 and CCL2 in several bladder cancer lung metastasis models [50]. Moreover, *ET-1* gene depletion, pharmacological inhibition of ET receptors, or transient macrophage depletion, blocked lung metastasis. These observations might validate the importance of the interplay between macrophages and tumor cells in lung metastatic process. In bladder cancer metastasis to lungs, the interaction can be regulated by the CCL2-CCR2 axis-regulated macrophages and tumor cells expressing [51]. Tumor cell-derived tissue factor induces clot formation, which can recruit CD11b^+^CX3CR1^+^ IM-like macrophages into lungs in mouse melanoma lung metastasis model [52]. Genetic or pharmacological inhibition of coagulation reduced macrophage recruitment and tumor cell survival. Moreover, tumor cell survival was decreased without altering clot formation in either *Mac1*-deficient mice or in *CD11b*-positive cell depleted mice, which displayed impaired macrophage functions, demonstrating the crucial role of functional macrophages in lung metastasis [52]. A subsequent study revealed that macrophage migration proceeded under the guidance of vascular cell adhesion molecule (VCAM)-1 and vascular adhesion protein (VAP-1) present on endothelial cells in these models [53]. Breast cancer cells frequently secreted Dickkopf-1 (DKK1), a signal transducer in Wnt pathway [54]. DKK1 inhibited breast cancer metastasis to lungs together with reduced macrophage and neutrophil infiltration, and impaired TGF-β expression. These activities were ascribed to DKK1-mediated non-canonical Wnt/PCP-RAC1-JNK and WNT/Ca^2+^-CaMKII-NF-κB signaling in cancer cells [54].

The abovementioned studies however, lacked mostly the analysis of surface markers in lung macrophages and therefore did not clearly discriminate between AMs, IMs, and macrophages derived from circulating monocytes.

A macrophage-tropic chemokine, CCL2, was produced by breast cancer cells in lungs, to recruit circulating monocytes expressing CCR2, a specific receptor for CCL2, to the tumor sites [16]. These monocytes represented classical monocytes and were differentiated into perivascular IMs, which mediated tumor extravasation into lung by releasing VEGF to increase vascular permeability. Pollard et al., proposed to designate them as MAMs [16]. The recruited MAMs secreted CCL3, which could promote MAM-cancer cell interactions and enhance subsequent seeding of cancer cells into lungs [55]. Moreover, VEGF-R1-expressing MAMs produced GM-CSF/CSF2 to promote metastatic growth of breast tumors in vivo [56]. Furthermore, MAMs exhibited immunosuppressive functions as evidenced by the suppressed cytotoxicity of CD8^+^ cytotoxic T cells, in part through superoxide production [57]. Nevertheless, MAMs seemed to differentiate into IMs in lungs since they expressed a characteristic IM marker, CD11b [16,55].

An intravenous injection of mouse lung carcinoma cells induced CCR2-independent local accumulation of IMs as well as CCR2-dependent recruitment of circulating monocytes into lungs [58]. Recruited monocytes acquired surface markers as resident IMs and could promote the spread of tumor in lungs, while resident IMs could support tumor growth. Similar cooperation between blood-derived macrophages and lung-resident macrophages was observed in lung metastasis models of mouse melanoma and breast cancer [59]. In these models, circulating tumor-derived microparticles entered the lung parenchyma readily and were taken up by both AMs and IMs, which subsequently produced CCL2 [59]. The produced CCL2 recruited CD11b^+^Ly6C^high^ inflammatory monocytes to the lungs, where the recruited cells matured into CD11b^+^Ly6C^−^ IM-like macrophages which produced IL-6 and triggered fibrin deposition, thereby providing the tumor cells with signals to support their survival and growth.

In addition to recruited monocytes and IMs, evidence is accumulating to indicate the roles of AMs in lung metastasis. Induction of lung inflammation by direct and specific NF-κB activation in airway epithelial cells enhanced lung metastasis arising from intravenous injection of Lewis lung carcinoma cells. On the contrary, depletion of AMs by intratracheal CLL injection, abrogated this enhanced metastasis [60]. However, no precise roles were assigned to AMs in this study.

Thymic stromal lymphopoietin (TSLP) was abundantly expressed by several mouse and human breast cancer cell lines. Tumor cell-derived TSLP induced AMs to express VEGF-A, platelet-derived growth factor-A, and MMP-9 thereby promoting lung metastasis [61]. In this model, intratracheal CLL injection reduced AMs but not IMs, together with reduced metastatic burden. These observations provided definitive evidence supporting the essential involvement of AMs in lung metastasis.

**Figure 4:**
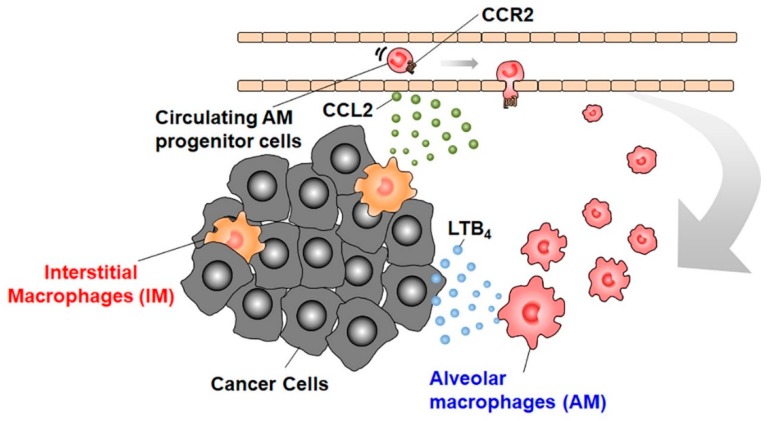
Interplay between IMs and AMs in lung metastasis. AMs are recruited from blood circulation, under the guidance of IM-derived CCL2 into metastatic lungs and contribute to the progression of lung metastasis by providing LTB_4_.

We recently observed that an intravenous injection of mouse hepatocellular carcinoma cells caused lung metastasis, together with increases in both F4/80^+^CD11b^−^CD11c^+^ AMs and F4/80^+^CD11b^+^CD11c^−^ IMs [39]. Intratracheal injection of CLL transiently reduced AMs but not IMs, and attenuated lung metastasis upon its administration at a later step when tumor cells had already seeded into lungs. In this metastatic model, AMs increased independently of cell proliferation while blood-borne CCR2-expressing AM precursors migrated from circulation into lungs by the action of CCL2 produced by IMs [39]. Migrated AM precursors matured to express F4/80 and CD11c, but not CD11b, the marker which is distinct for MAMs [16]. These AMs abundantly expressed 5-lipoxygenase (5-LOX), to generate leukotriene (LT) B_4_, the molecule which could promote tumor cell growth in lungs. 5-LOX-positive AMs were increased in metastatic lungs of HCC mice, suggesting that similar mechanisms might also operate in human lung metastasis [39]. Thus, lung metastasis might proceed through the dynamic interaction between IMs and AMs (Figure 4), although this assumption warrants further studies.

## 4. Strategies to Use Intrapulmonary Macrophages as a Weapon for Lung Metastasis

The crucial involvement of TAMs in various aspects of tumor progression, makes them a good target for anti-cancer therapy. Three strategies are proposed to target TAMs in cancer therapy; depletion of TAMs, inhibition of TAM recruitment, and reprogramming of TAMs. In this section, we will outline these three strategies with focusing mainly on lung metastasis, and for further information, the readers are recommended to refer to more extensive reviews [62,63].

As macrophages depend on GM-CSFR/CSF2R signaling, different antibodies and small molecules targeting GM-CSFR/CSF2R are used to deplete TAMs in various situations. These agents are effective for some types of cancers, particularly those overexpressing GM-CSF/CSF2, but frequently cause severe adverse effects, thereby limiting dose escalation [64]. Bisphosphonates also deplete TAMs, thereby promoting sorafenib-mediated attenuation of lung metastasis arising from orthotopic injection of human hepatocellular carcinoma cells into nude mice [65]. Moreover, bisphosphonates can prolong overall survival of postmenopausal patients with breast cancer in an adjuvant setting [66]. However, it remains elusive whether this outcome is due to their effects on metastasis, particularly in the lungs.

A substantial portion of TAMs are derived from CCR2-expressing circulating classical monocytes, which migrate to the tumor sites under the guidance of tumor cell-derived CCL2 [62]. Therefore, blocking the CCL2-CCR2 axis is proposed to inhibit TAM recruitment. Indeed, CCL2 neutralization in mice inhibited lung metastasis in four syngeneic mouse models of metastatic breast cancer, by retaining monocytes in the bone marrow [67]. However, subsequent interruption of CCL2 inhibition released monocytes from the bone marrow and enhanced cancer cell mobilization from the primary sites, blood vessel formation and metastatic cell proliferation in the lungs in an IL-6- and VEGF-dependent manner, thereby resulting in an increase of lung metastasis. Moreover, tissue-resident macrophages also constitute TAMs and can support tumor progression more efficiently than circulating monocyte-derived TAMs, in mouse pancreatic duct adenocarcinoma [68]. Thus, it is likely that the inhibition of circulating monocyte recruitment could be overcome by compensatory proliferation of tissue-resident macrophages. These phenomena might account for the largely disappointing clinical trials targeting the CCL2-CCR2 axis [62].

Macrophage polarization is presumed to be flexible and the reversion from M2 to M1 phenotype can confer TAMs with cytotoxic activity [21]. Based on these assumptions, several measures have been proposed to enhance anti-tumor activity of intrapulmonary macrophages by reprogramming lung macrophage polarization from an M2- to an M1-like phenotype with potent cytotoxic activity.

In a tail vein metastasis model with mammary tumor cell lines established from mouse mammary tumor virus-polyoma middle T-antigen-derived tumors, NF-κB activation in lung macrophages induced their reversion into an M1-phenotype with enhanced production of reactive oxygen species and increased tumor cell apoptosis, thereby reducing lung metastases, when it was activated at tumor cell colonization in lungs [69]. Anti-PD-1 treatment led to a significant reduction in lung metastasis model of osteosarcoma, together with enhanced NK cell and macrophage infiltration into metastatic sites [70]. However, the therapeutic effects were reduced by depletion of intrapulmonary macrophages, but not NK cells. Moreover, anti-PD-1 therapy redirected macrophages from an M2- to an M1-like phenotype [70]. Paradoxically, intratracheal administration of IL-10 suppressed lung tumorigenesis caused by activated K-ras^G12D^ mutation, together with a dramatic M1 to M2 phenotypic change in IMs [71]. M2-polarized IMs suppressed Th17 functions, which is critical for lung tumorigenesis.

Repeated administration of aerosolized IFN-γ markedly reduced lung metastasis arising from subcutaneous inoculation of Lewis lung carcinoma cells and enhanced anti-proliferative activities of intrapulmonary macrophages [72]. Liposomes were prepared by incorporating a lipopeptide analogue of a fragment from the cell wall of gram-negative bacteria. Systemic administration of these liposomes enhanced the cytotoxicity of AMs and reduced the incidence of spontaneous lung metastasis in mice whose primary renal adenocarcinoma was removed by nephrectomy [73,74]. Likewise, muramyl tripeptide was encapsulated into phosphatidylcholine liposomes, which upon oral administration could be rapidly absorbed in the intestine and reach the systemic circulation [75]. Repeated oral administration of the liposomes sustained enhanced cytotoxic activities of AMs, together with augmented capacity to secrete TNF-α and IL-6 and inhibited lung metastasis arising from an intravenous injection of a renal cancer cell line [75].

The administration of aerosolized Toll-like receptor (TLR)9 and TLR3 agonists reduced lung metastasis arising from an intravenous injection of melanoma cells, in an NK-dependent manner [76]. In this setting, NK cell activation required AMs pretreated with TLR9/3 agonists and was reciprocally able to polarize naïve AMs toward a M1-like phenotype with enhanced IL-12 expression [77]. 

Exosomes from poorly metastatic mouse melanoma cells expanded Ly6C^low^ patrolling monocytes in bone marrow and eventually recruited patrolling monocytes and NK cells, thereby killing melanoma cells in a TNF-related apoptosis-inducing ligand (TRAIL)-dependent manner [78]. Similarly, exosomes isolated from patients with non-metastatic primary melanomas had the ability to suppress lung metastasis. These observations suggest that exosomes from patients with non-metastatic cancer, could be utilized to prevent and/or treat lung metastasis.

## 5. Future Perspective

Recent studies have substantially promoted the understanding of the ontogeny, phenotypes, and functions of lung macrophage and monocyte populations, under steady or inflammatory conditions. However, there are still unanswered questions regarding the mechanisms underlying their trafficking and their phenotypic and functional polarization in lung metastatic sites. Moreover, the cellular and molecular mechanisms underlying the interactions between lung macrophages/monocytes and other resident stromal cells including endothelial cells, fibroblasts, and other hematopoietic cells such as neutrophils, remains elusive. Furthermore, the precise role of lung macrophages/monocytes has not yet been determined in terms of local tumor immunity, particularly innate immune memory. The elucidation of these points is required to develop novel therapeutic measures against lung metastasis by targeting these cell populations.

## Figures and Tables

**Table 1:** Major surface markers of mouse and human lung monocyte/macrophage populations [11,12].

Surface Markers	AMs	IMs	Classical Monocytes	Patrolling Monocytes
Mouse markers				
F4/80	+	+	+/low	−
CCR2	−	low	+	low
CX3CR1	−	+	+	+
CD11b	−	+	+	+
CD11c	+	−/low	−	−
CD64	+	+	−	−
CD86	+	+	low	low
CD169	+	low	−	−
Ly6C	−	low	+	low
MHC class II	low	+	−	−
SiglecF	−	−	−	−
Human markers				
CD11b	+	+	+	+
CD11c	+	+	undetermined	undetermined
CD14	−	+	+	+
CD16	+	intermediate	−	+
CD169	+	−	−	−
CD206	+	intermediate	−	−
CD45	+	+	+	+
CD64	+	+	+	+
CD71	+	low	low	low
CD80	+	low	−	−
CD86	+	low	−	−
HLA-DR	+	+	+	+

## Abbreviations

AMs	alveolar macrophages
CEBPβ	CCAT/enhancer binding protein β
CLL	clodronate liposome
CD	cluster of differentiation
CSF	colony stimulating factor
COX	cyclooxygenase
DC	dendritic cell
DKK	Dickkopf
ET	endothelin
EMT	epithelial mesenchymal transition
GM	granulocyte-macrophage
HLA	human leukocyte antigen
HIF	hypoxia-inducible factor
IFN	interferon
IL	interleukin
IMs	interstitial macrophages
LT	leukotriene
LOX	lipoxygenase
MHC	major histocompatibility antigen
MMP	matrix metalloproteinase
MAMs	metastasis-associated macrophages
NK	natural killer
NO	nitric oxide
PPAR	peroxisome proliferator-activated receptor
ROS	reactive oxygen species
R	receptor
TLR	toll-like receptor
TGF	transforming growth factor
TAMs	tumor-associated macrophage
TSLP	thymic stromal lymphopoietin
TNF	tumor necrosis factor
TRAIL	TNF-related apoptosis-inducing ligand
VAP	vascular adhesion protein
VCAM	vascular cell adhesion molecule
VEGF	vascular endothelial growth factor
VHL	von Hippel Lindau protein
